# Multiple-Timescale Neural Networks: Generation of History-Dependent Sequences and Inference Through Autonomous Bifurcations

**DOI:** 10.3389/fncom.2021.743537

**Published:** 2021-12-10

**Authors:** Tomoki Kurikawa, Kunihiko Kaneko

**Affiliations:** ^1^Department of Physics, Kansai Medical University, Hirakata, Japan; ^2^Department of Basic Science, Graduate School of Arts and Sciences, University of Tokyo, Tokyo, Japan; ^3^Center for Complex Systems Biology, Universal Biology Institute, University of Tokyo, Tokyo, Japan

**Keywords:** slow-fast systems, recurrent neural networks, bifurcations, sequential patterns, non-Markov sequences

## Abstract

Sequential transitions between metastable states are ubiquitously observed in the neural system and underlying various cognitive functions such as perception and decision making. Although a number of studies with asymmetric Hebbian connectivity have investigated how such sequences are generated, the focused sequences are simple Markov ones. On the other hand, fine recurrent neural networks trained with supervised machine learning methods can generate complex non-Markov sequences, but these sequences are vulnerable against perturbations and such learning methods are biologically implausible. How stable and complex sequences are generated in the neural system still remains unclear. We have developed a neural network with fast and slow dynamics, which are inspired by the hierarchy of timescales on neural activities in the cortex. The slow dynamics store the history of inputs and outputs and affect the fast dynamics depending on the stored history. We show that the learning rule that requires only local information can form the network generating the complex and robust sequences in the fast dynamics. The slow dynamics work as bifurcation parameters for the fast one, wherein they stabilize the next pattern of the sequence before the current pattern is destabilized depending on the previous patterns. This co-existence period leads to the stable transition between the current and the next pattern in the non-Markov sequence. We further find that timescale balance is critical to the co-existence period. Our study provides a novel mechanism generating robust complex sequences with multiple timescales. Considering the multiple timescales are widely observed, the mechanism advances our understanding of temporal processing in the neural system.

## 1. Introduction

Sequentially activated patterns are widely observed in neural systems, for instance, the cerebral cortex (Jones et al., 2007; Ponce-Alvarez et al., 2012; Stokes et al., 2013; Mazzucato et al., 2015; Kurikawa et al., 2018; Taghia et al., 2018), hippocampus (HPC) (Gupta et al., 2010; Maboudi et al., 2018; Schuck and Niv, 2019; Wimmer et al., 2020), and the striatum (Akhlaghpour et al., 2016). These patterns underlie a range of cognitive functions: perception (Jones et al., 2007; Miller and Katz, 2010), decision making (Ponce-Alvarez et al., 2012), working memory (Stokes et al., 2013; Taghia et al., 2018), and recall of long-term memory (Wimmer et al., 2020). They process temporal information by concatenating shorter sequences (Gupta et al., 2010), reorganizing the order in sequential patterns (Wimmer et al., 2020), and chunking sequences (Jin et al., 2014), which lead to inference and recall, based on previous experiences.

Several models have been proposed to understand how such sequential patterns are shaped in the neural systems to perform complex tasks (Kleinfeld, 1986; Sompolinsky and Kanter, 1986; Seliger et al., 2003; Gros, 2007; Sussillo and Abbott, 2009; Russo and Treves, 2012; Laje and Buonomano, 2013; Recanatesi et al., 2015; Chaisangmongkon et al., 2017; Haga and Fukai, 2019). Popular Hebbian models provide a simple framework in which each pattern in the sequence is represented as a metastable state, which is formed through Hebbian learning. An asymmetric connection from the current to the successive pattern (Amari, 1972; Kleinfeld, 1986; Sompolinsky and Kanter, 1986; Nishimori et al., 1990; Seliger et al., 2003; Gros, 2007; Russo and Treves, 2012; Recanatesi et al., 2015; Haga and Fukai, 2019) causes transition between patterns. Such transitions are also induced by slower destabilization terms (Gros, 2007; Russo and Treves, 2012; Recanatesi et al., 2015). Note that these sequences are widely observed in neural systems (Miller, 2016). In other studies (Sussillo and Abbott, 2009; Laje and Buonomano, 2013; Mante et al., 2013; Chaisangmongkon et al., 2017), recurrent neural networks (RNN) are trained by using machine learning methods so that experimentally observed neural dynamics are generated.

Despite the great success of these studies, however, some fundamental questions remain unanswered. In models that generate sequential metastable states, a transition between these states is embedded rigidly into the connectivity (i.e., the correlation between the current to the next pattern), resulting in successive patterns being determined by the immediately preceding pattern. Hence, the generation of sequences depending on the long history of the previous patterns is not possible. On the other hand, RNNs trained with machine learning methods allow for generating complex sequences dependent on history. The training methods require non-local information and have to retain the information until the sequence finishes, which is not biologically plausible. In addition, the formed sequences are vulnerable to noise or perturbation to the initial state (Laje and Buonomano, 2013).

**Figure 1:**
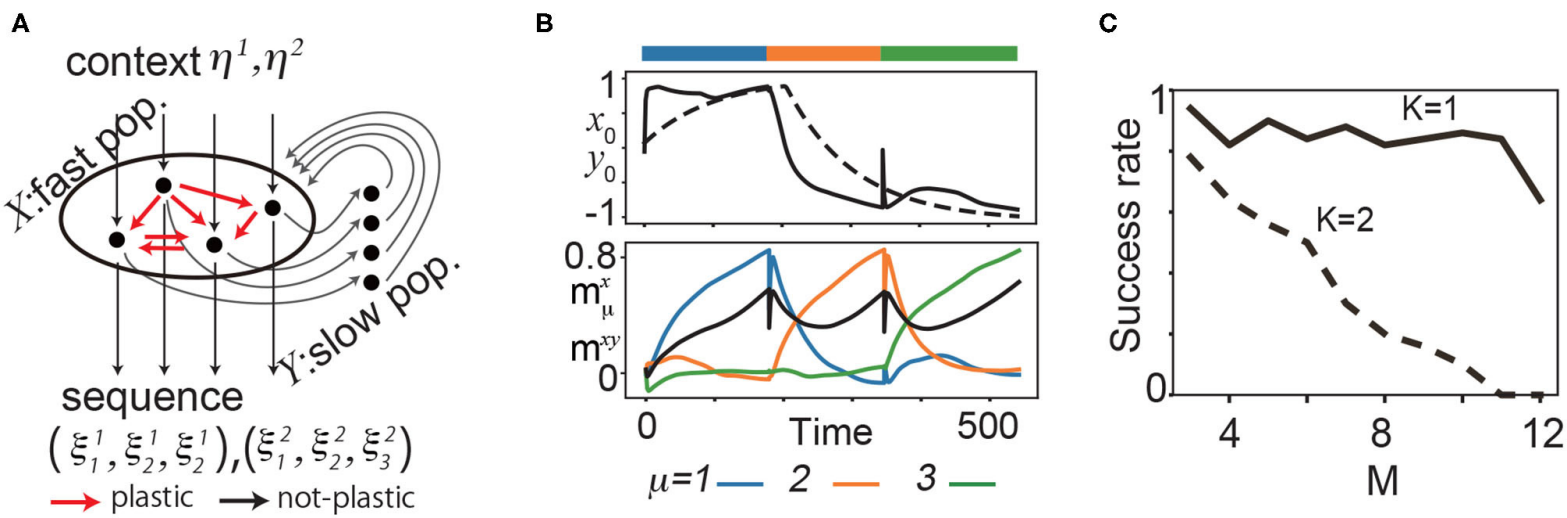
**(A)** Schematic diagram of the proposed model for two sequences (*K* = 2) and three patterns (*M* = 3). **(B)** Neural dynamics during the learning process of three targets. Top: the time series of one of the fast variables *x*_0_ (solid line) and the corresponding slow variable *y*_0_ (broken line) during the learning process. Bottom: $m_{1,2,3}^{x}$, overlaps of ***x*** with $\xi_{1}^{1}$ (blue), $\xi_{2}^{1}$ (orange), and $\xi_{3}^{1}$ (green). The black line represents the overlap between ***x*** and ***y*** denoted as *m*^*xy*^. The bars above the panels indicate the targeted patterns given to the network in corresponding periods. **(C)** The fraction of successful recalls is plotted as a function of *M* for *K* = 1, 2. It is averaged over 50 realizations (10 networks and five pairs of the target and input patterns for each network). Here, a successful recall is defined as the case in which all *K* × *M* targets are sequentially generated in the correct order in the presence of the corresponding inputs.

To resolve these unanswered questions, we introduce a neural network model with slow and fast neurons that can learn the history-dependent sequences and connect the sequences. The fast neural dynamics generate patterns in response to an external input with the feedback from the slow dynamics. The slow dynamics store the history of the inputs *via* the fast dynamics, and feed the stored information back to the fast, as shown in Figure 1A. By this model, we provide a novel framework in temporal processing in the neural system in which the slow dynamics control successive bifurcations of fixed points of fast dynamics, based on the stored history of previous patterns and inputs. By adopting a biologically plausible learning rule based solely on the correlation between the pre- and post-synaptic neural activities as introduced previously (Kurikawa and Kaneko, 2013, 2016; Kurikawa et al., 2020), we demonstrate that our model with the fast and slow neural dynamics memorizes the history-dependent sequences and enables inference based on them.

Multiple-timescale neural dynamics are observed across cortical areas (Honey et al., 2012; Murray et al., 2014; Chaudhuri et al., 2015; Hasson et al., 2015). Neural activities in lower sensory cortices change in a faster timescale and respond instantaneously to stimuli, whereas those in higher association cortices change in a slower timescale and integrate information over longer periods. Cooperations between the higher and lower cortices are necessary to process the temporal information.

Some model studies focused on the multiple-timescale dynamics (Kiebel et al., 2008; Yamashita and Tani, 2008) and showed that their models generate history-dependent sequences. These models, however, adopted machine learning methods, and thus, biological plausibility is hard to be assured. In contrast, our model proposes a biologically plausible mechanism in which the higher cortices regulate the lower ones to generate complex sequences.

In the following, we focus on two basic aspects of neural sequences in temporal information processing; context (history)-dependent sequences and inference. In the context-dependent working memory task (Mante et al., 2013; Stokes et al., 2013), distinct sequences of neural patterns are evoked by identical stimuli depending on the preceding context signals. Second, in this study, inference is defined as the ability to make appropriate responses against a new environment by using previously learned examples. For instance (Jones et al., 2012; Wikenheiser and Schoenbaum, 2016), consider a rat learning successive stimuli, A followed by B, and then reward C. After changing the environment, the rat is required to learn a new combination of stimuli, A' followed by B. In this situation, the rat is able to infer that stimuli A' causes the reward C *via* B. Neural activities reflecting this cognitive function should show sequential patterns A'BC even after learning only A'B. After showing basic behaviors in our model, we demonstrate that how such a context-dependent sequence is generated and how inference is executed.

## 2. Materials and Methods

### 2.1. Neural Model

We consider learning of *K* sequences, each of which contains *M* patterns, with *K* input patterns. We denote the μ-th targeted pattern in the α-th sequence as $\xi_{\mu }^{\alpha }$, and the corresponding input as ***η***^α^ for μ = 1, 2, ⋯ , *M* over the inputs α = 1, ⋯ , *K*. Figure 1A illustrates the case with *K* = 2 and *M* = 3: In this case, a given sequence $\xi_{1}^{\alpha }$,$\xi_{2}^{\alpha }$,$\xi_{3}^{\alpha }$ (α = 1, 2) should be generated upon a given corresponding input ***η***^α^. Generally, a pattern to be generated next is determined not only by the current pattern but also by earlier patterns. Thus, a network has to retain the history of previous patterns to generate a sequence correctly.

To achieve this, we built a two-population model with different timescales, one with *N* fast neurons and one with *N* slow neurons, denoted as *X* and *Y*, respectively. *X* receives an external input, and *Y* receives the output from *X* and provides input to *X*, as shown in Figure 1A. The neural activities *x*_*i*_ in *X* and *y*_*i*_ in *Y* evolve according to the following equation:


(1) $$\begin{array}{r} \tau_{x}\overset{∙}{x_{i}}=\tanh\left(\beta_{x}I_{i}\right)-x_{i}, \end{array}$$

(2) $$\begin{array}{r} \tau_{y}\overset{∙}{y_{i}}=\tanh\left(\beta_{y}x_{i}\right)-y_{i}, \end{array}$$

(3) $$\begin{array}{r} I_{i}=u_{i}+\tanh\left(r_{i}\right)+{\left(\eta^{\alpha }\right)}_{i}, \end{array}$$

where $u_{i}=\overset{N}{\underset{j\neq i}{\sum }}J_{ij}^{X}x_{j}$; $r_{i}=\overset{N}{\underset{j}{\sum }}J_{ij}^{XY}\tanh\left(y_{j}\right)$. $J_{ij}^{X}$ is a recurrent connection from the *j*-th to the *i*-th neuron in *X*, and $J_{ij}^{XY}$ is a connection from the *j*-th neuron in *Y* to the *i*-th neuron in *X*. *J*^*X*^ is a fully connected network without self connections. It is modified during the learning process as described in the following subsection “Learning model” and initialized with the binary values $P\left[J_{ij}^{X}=\pm {\left(N-1\right)}^{-1/2}\right]=1/2$. The diagonal entries of *J*^*X*^ are kept at zero during the entire learning process. *J*^*XY*^, in contrast, is a non-plastic sparse network; $P\left(J_{ij}^{XY}=\pm cN^{-1/2}\right)=\rho$ and $P\left(J_{ij}^{XY}=0\right)=1-2\rho$. *X* is required to generate the pattern $\xi_{\mu }^{\alpha }$ in the presence of ***η***^α^, i.e., an attractor that matches $\xi_{\mu }^{\alpha }$ is generated under ***η***^α^. The *i*-th element of a targeted pattern denoted as ${\left(\xi_{\mu }^{\alpha }\right)}_{i}$, is assigned to the *i*-th neuron in *X*, and randomly sampled according to the probability $P\left[{\left(\xi_{\mu }^{\alpha }\right)}_{i}=\pm 1\right]=1/2$. The input ${\left(\eta^{\alpha }\right)}_{i}$ is injected to the *i*-th neuron in *X*, randomly sampled according to $P\left[{\left(\eta^{\alpha }\right)}_{i}=\pm 1\right]=1/2$. ***ξ*** and ***η*** are the same dimensional vectors as the fast dynamics, i.e., *N*-dimensional vectors. We set *N* = 100, β_*x*_ = 2, β_*y*_ = 20, τ_*x*_ = 1, τ_*y*_ = 100, ρ = 0.05, and *c* = 7. The dependence of the performance on these parameters are shown in the Supplementary Materials, while the details for the parameter setting are described.

In Equation (3), we implement the non-linear activation at the apical dendrites (Larkum et al., 2009). We assumed that the input from *Y* to *X* innervated on the apical dendrites of neurons in *X*, which is consistent with observations that the feedback inputs from the higher cortical areas innervate on the apical dendrites of layer 5 pyramidal neurons (Larkum, 2013), whereas the recurrent input from *X* to *X* was assumed to innervate the proximal dendrites. The synaptic inputs to the apical dendrites are integrated and evoke the calcium spike when the integrated input exceeds the threshold of spikes (refer to the detail of this type of spike in Larkum et al., 2009). To reproduce this information processing at the apical dendrites, we used two nonlinear filters by the hyperbolic tangent function for the input from *Y* to *X*. First, by adopting tanh(*y*_*j*_), the activity of the *j*-th neuron in *Y* is amplified in a nonlinear way at a synapse onto the neuron *x*_*i*_. Second, tanh(*r*_*i*_) represents calcium spike at the branching point of the tuft dendrite. Even if these hyperbolic tangent functions are not leveraged in Equation (3), the behavior of the model is not changed qualitatively, although the performance of the model is reduced.

### 2.2. Learning Model

Only *J*^*X*^ changes to generate the target according to the following equation:


(4) $$\begin{array}{r} \tau_{syn}\overset{∙}{J_{ij}^{X}}=\left(1/N\right)\left(\xi_{i}-x_{i}\right)\left(x_{j}-u_{i}J_{ij}^{X}\right), \end{array}$$

where τ_*syn*_ is the learning speed (set to 100). This learning rule comprises a combination of a Hebbian term between the target and the presynaptic neuron, and an anti-Hebbian term between the pre- and post-synaptic neurons with a decay term $u_{i}J_{ij}^{X}$ for normalization ^1^. This form satisfies locality across connections and is biologically plausible (Kurikawa et al., 2020). We previously applied this learning rule to a single network of *X* and demonstrated that the network learns *K* maps between inputs and targets, i.e., *M* = 1 (Kurikawa and Kaneko, 2013, 2016; Kurikawa et al., 2020). However, in that case, generating a sequence (*M*≥2) was not possible. In the present study, there are two inputs for *X*, one from input ***η*** and one from *Y* that stores previous information. Thus, the network can generate a pattern depending not only on the present input pattern, but also on the previous patterns.

### 2.3. Learning Procedure

In our model, the patterns in the sequence are learned sequentially. A learning step of a single pattern is accomplished when the neural dynamics satisfy the following two criteria: ***x*** sufficiently approaches the target pattern, i.e., $m_{\mu }^{x}\equiv \Sigma_{i}x_{i}{\left(\xi_{\mu }^{1}\right)}_{i}/N>0.85$, and ***y*** is sufficiently close to ***x***, i.e., Σ_*i*_*x*_*i*_*y*_*i*_/*N*>0.5. After the completion of one learning step, a new pattern $\xi_{2}^{1}$ is presented instead of $\xi_{1}^{1}$ with a perturbation of fast variables *x*_*i*_, by multiplying a random number uniformly sampled from zero to one. We execute these steps sequentially from μ = 1 to *M* to learn a sequence once, denoted as one epoch of the learning. Before finishing the learning process, this procedure is repeated 20 times (i.e., 20 epochs). The second criterion for terminating the learning step is introduced for memorizing the sequences, especially the history-dependent sequences. Further, the value 0.5 of this criterion must take an intermediate value. If this criterion is not adopted or this criterion value is small, the target pattern is switched as soon as ***x*** is close to the target during the learning process. At this time, ***y*** is far from ***x*** because ***y*** is much slower than ***x***. In this case, ***y*** cannot store any information about ***x***. On the other side, when the value is close to unity, ***y*** matches ***x*** and ***y*** can store only the present ***x***. In both cases, ***y*** cannot store the history of ***x***.

#### 2.3.1. Inference Task

In the inference task, we present sequentially different inputs in a sequence, whereas a single input is applied for a sequence in other tasks. We include the super- and sub-scripts in the notation of ***η*** as $\eta_{\mu }^{\alpha }$ that represents the μ-th input pattern in the α-th sequence. In this task, a network learns three sequences:(*S, A, B, C*), (*S, A*′, *B, C*), and (*D*). The former two sequences are used for inference, whereas the last one is a distractor to prevent the over-stability of the other two sequences. First, in the learning process, the network learns (*S, A, B, C*) and (*D*). Second, it learns (*S, A*′, *B*) after training is completed. Then, we examine if the network generates (*S, A*′, *B, C*), implying inference from *B* to *C*.

For the first sequence (*S, A, B, C*), we apply $\eta_{1}^{1}=s$ for the target *S*, $\eta_{2}^{1}=a$ for *A*, and $\eta_{3}^{1}=\eta_{4}^{1}=b$ for *B* and *C*. For the second sequence (*A*′, *B, C*), we apply $\eta_{1}^{1}=s$ for the target *S*, $\eta_{2}^{2}=a^{\prime }$ for *A*′, and $\eta_{3}^{2}=b$ for *B*. All the targets and inputs are randomly sampled according to the probability $P\left[{\left(\xi_{\mu }^{\alpha }\right)}_{i}=\pm 1\right]=P\left[{\left(\eta_{\mu }^{\alpha }\right)}_{i}=\pm 1\right]=1/2$.

Because, in this task, the input pattern is changed in a single sequence, we apply the input and target over 100 unit times and change them to the next in the sequence through the learning process. In the recall process, we regularly change the input pattern every 100 unit times, independently of the value of the neural activities.

### 2.4. Data Analysis

#### 2.4.1. Principal Component Analysis (PCA)

**Figure 2:**
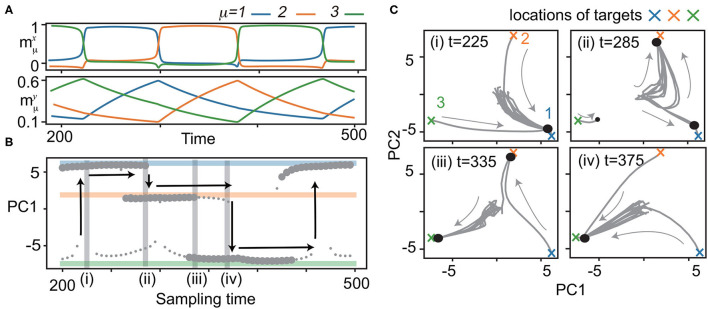
Bifurcation of ***x*** with quenched ***y***. **(A)** Neural dynamics during the recall process of the three learned patterns. Overlaps of neural activities $m_{\mu }^{x,y}$, μ = 1, 2, 3 in ***x*** (top) and ***y*** (bottom) for *M* = 3 are plotted in the same color as shown in Figure 1B. ***y*** is sampled from the trajectory at 200 < *t* <500 for the bifurcation diagram of ***x*** shown in **(B)**. **(B)** Bifurcation diagram of ***x*** as quenched ***y*** is updated with the sampling time. Fixed points of ***x*** are shown by projecting to the first principal component (PC1) of principal component analysis (PCA). Small circles indicate fixed points with small basins: neural activity beginning only from the vicinity of the target converges to these points. Large circles represent fixed points with large basins: neural activities from the initial states converge to these points. To identify fixed points, the neural states are plotted after the transient period. Colored lines indicate the locations of the targets ($\xi_{1,2,3}^{1}$ in blue, orange, and green, respectively). Vertical arrows show the transitions of ***x*** to different targets in the recall process. **(C)** The neural dynamics for a given ***y*** at *t* = 225, 285, 335, 375 shadowed in **(B)** are depicted by projecting ***x*** to the 2-dimensional principal component (PC) space [PC1 is same as that in **(B)**]. Fifteen trajectories (three from the vicinity of the target, and others from random initial states) are plotted. Large and small circles represent fixed points given in **(B)**.

We analyzed neural trajectories by using mainly PCA in Figures 2, **5**–**7** and Supplementary Figure S5. The *N* × *T* dimensional neural data of ***x*** is used for the PCA. In this study, *T* is the duration time to analyze neural dynamics multiplied by the sampling number of ***x*** per unit time (In this case, 20). For analysis of *y*, we also used PCs obtained by x.

#### 2.4.2. Calculation of Success Rate in the Inference Task

To compute the success rate of the generation of the sub-sequence (*B, C*) under input *b*, we first identify the sequence of the patterns from the continuous neural activity by setting a threshold for the overlap value at 0.7. In the recall process, since the overlap with either (or a few) of the targets *S, A, B, C, D, A*′ is selectively high most of the time, the sequences composed of some of the patterns *S, A, B, C, D, A*′ are obtained for each of different input sequences (*s, a, b, b*), (*s, a*′, *b, b*), and (*s, v, b, b*). In this study, *v* is a random pattern that is not used for learning. We generate these sequential patterns starting from 20 initial states for each of 100 network realizations and use the subpart of them in the presence of *b* to calculate the success rate of the sub-sequence (*B, C*). In this study, 100 network realizations are obtained by generating *J*^*X*^, *J*^*XY*^, ***ξ***, and **η** 100 times according to independent and identically probability distributions as described in the “Neural model” in this section.

We test significant differences in the success rates of the generation of the sub-sequence (*B, C*) for different input sequences by Wilcoxon's signed-rank test. The success rates for (*s, a*′, *b*) and (*s, v, b*) are calculated from 20 sequences for each network realization. Hundred samples of two related paired success rates are obtained and used in Wilcoxon's signed-rank test.

## 3. Results

Before exploring the history-dependent sequence, we analyzed if our learning rule generates simple sequences, namely, sequences in which the successive pattern is determined solely by the current pattern. Figure 1B shows a sample learning process for *K* = 1. We applied ***η***^1^ to a network and presented $\xi_{1}^{1}$ as the first pattern of a target sequence. After the transient time, ***x*** converges to $\xi_{1}^{1}$ due to synaptic change. ***y*** follows ***x*** according to Equation 2 and, consequently, moves to the target.

We present an example of a recall process after the learning process for (*K, M*) = (1, 3) in Figure 2A. In recall, the connectivity is not changed. The initial states of the fast variables are set at random values sampled from a uniform distribution of –1 to 1. The slow variables are set at values of their final states in the learning process in order for the network to generate the sequence starting from $\xi_{1}^{1}$. (If the slow variables are set randomly in a similar manner as the fast variables, the network can still generate the sequence, but the first pattern of the sequence is not $\xi_{1}^{1}$). The targets appear sequentially in *X* in order. Note that in the recall process, the transition occurs spontaneously without any external operation.

We explored the success rate of the learning and found that increasing *M* and *K* generally leads to a decrease in the success rate of recalls. For *N* = 100 and *K* = 1, the success rate is over 80% for *M* = 1 up to 11, and decreases beyond *M* = 12. For *K* = 2, the success rate is approximately 80% for *M* = 3 and decreases gradually as *M* increases (Figure 1C, refer to the Supplementary Material for detailed results). Furthermore, we investigated how the balance between the timescale of the slow variables τ_*y*_ and that of learning τ_*syn*_ affect the success rate.

**Figure 3:**
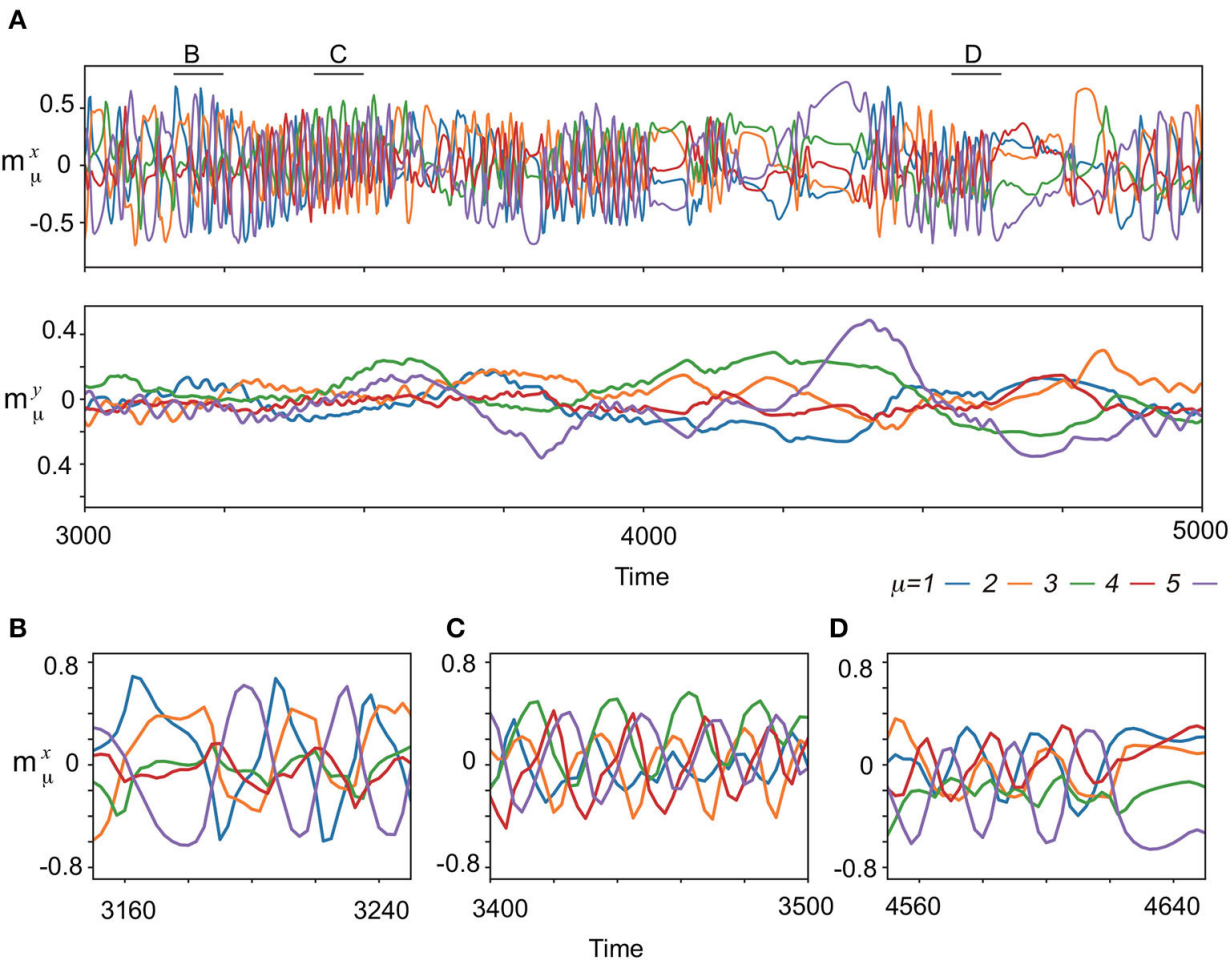
The neural dynamics without input after the learning process for (*K, M*)=(1,5). **(A)** (Upper) Overlaps of the spontaneous fast dynamics with the targets $m_{\mu }^{x}$, μ = 1, 2, 3, 4, 5 are plotted in different colors indicated at bottom of the panels. (Lower) Overlaps of the spontaneous slow dynamics $m_{\mu }^{y}$, μ = 1, 2, 3, 4, 5 are plotted in the same colors as the top panel. **(B–D)** The enlarged view of spontaneous dynamics $m_{\mu }^{x}$ in **(A)** are shown. The enlarged time span adopted in each panel is indicated by the corresponding black bar at the top of **(A)**.

Next, the spontaneous activities without the input are analyzed. Figure 3A exemplifies the characteristic behavior of the complex spontaneous dynamics: fast oscillating activities and slowly varying ones appear alternatively. In the period of the oscillating activities, the memorized patterns are activated sequentially, which are not all memorized patterns, but their subsets, as shown in Figures 3B–D. Different subsets of patterns appear intermittently. For instance, (***ξ*^1^**, ***ξ*^2^**, ***ξ*^5^**), (***ξ*^3^**, ***ξ*^4^**, ***ξ*^5^**), and (***ξ*^1^**, ***ξ*^4^**, ***ξ*^5^**) are observed in Figures 3B–D, respectively. In contrast, in the period of the slowly varying activities, one or few patterns are stable for a while and then are collapsed.

### 3.1. Bifurcations of Fast Neural Dynamics

To elucidate how such a recall is possible, we analyzed the phase space of ***x*** with ***y*** quenched. In other words, ***y*** is regarded as bifurcation parameters for the fast dynamics. Specifically, we focused on the neural dynamics for 200 ≤ *t* ≤ 500, as shown in Figure 2A. In this period, the fast dynamics show transitions from $\xi_{1}^{1}$ to $\xi_{2}^{1}$ at *t* = 290, from $\xi_{2}^{1}$ to $\xi_{3}^{1}$ at *t* = 375, and from $\xi_{3}^{1}$ to $\xi_{1}^{1}$ at *t* = 220, 460. We sampled the slow variables every five units of time from *t* = 200 to 500, ***y***_*t* = 200_, ***y***_*t* = 205_, ⋯ , ***y***_*t* = 500_, along the trajectory, and analyzed the dynamics of ***x*** with the slow variables quenched at each sampled ***y***_*t* = 200, 205, ⋯ , 500_. Figure 2B shows the bifurcation diagram of ***x*** against the change in ***y***, and Figure 2C shows the trajectories of ***x*** for specific ***y***.

We now consider the neural dynamics for ***y***_*t* = 225_, just after the transition from $\xi_{3}^{1}$ to $\xi_{1}^{1}$ [Figure 2C (i)]. For this ***y***, a single fixed point corresponding to the present pattern ($\xi_{1}^{1}$) exists, leading to its stability against noise. As ***y*** is changed, the basin of $\xi_{1}^{1}$ shrinks, while a fixed point corresponding to the next target $\xi_{2}^{1}$ appears, and its basin expands ^2^, as shown in Figure 2C (ii). At ***y***_*t* = 290_, the fixed point $\xi_{1}^{1}$ becomes unstable. Thus, the neural state ***x*** at $\xi_{1}^{1}$ goes out of there, and falls on $\xi_{2}^{1}$, i.e., a transition occurs.

With a further shift of ***y***, ***y***_*t* = 295, 300, ⋯_, a regime of coexistence of $\xi_{2}^{1}$ and $\xi_{3}^{1}$ with large basins appears [Figure 2C (iii)]. The basin of the attractor $\xi_{2}^{1}$ shrinks and vanishes [Figure 2C (iv)], and the transition from $\xi_{2}^{1}$ to $\xi_{3}^{1}$ occurs at *t* = 375. The next transition from $\xi_{3}^{1}$ to $\xi_{1}^{1}$ occurs in the same manner at *t* = 460. These processes provide the mechanism for robust sequential recall: fixed points ***x*** of the current and successive targets coexist, and then, the current target becomes unstable when the slow variables change.

To examine the robustness of the recall, we explored trajectories from different initial conditions with Gaussian white noise with strength *s* (refer to Supplementary Material for details). All of these trajectories converge correctly to a target sequence after some transient period for weak noise. By increasing the noise strength, the recall performance of noisy dynamics is made equal to that of the noiseless dynamics up to noise strength *s* = 0.3. For stronger noise, the duration of residence at the target is decreased, because the neural state of ***x*** is kicked out of the target earlier than in the noiseless case. Even upon applying a strong and instantaneous perturbation to both ***x*** and ***y***, the trajectory recovers the correct sequence. The sequence is represented as a limit cycle containing ***x*** and ***y*** and, thus, is recalled robustly.

### 3.2. Inference by Concatenation

Next, we test if our model flexibly infers new sequences based on the previously learned sequence. To this end, we consider the following task (Refer to Materials and methods for details). First, a network learns a sequence $\left(\xi_{2}^{1},\xi_{3}^{1},\xi_{4}^{1}\right)=\left(A,B,C\right)$ in response to the associated input sequences $\left(\eta_{2}^{1},\eta_{3}^{1},\eta_{4}^{1}\right)=\left(a,b,b\right)$. In addition, we provide $\eta_{1}^{1}=s$ associated with $\xi_{1}^{1}=S$ preceding the sequence as a fixation cue and the response to it (e. g., the subject's gaze to the fixation point), respectively. After the learning is completed, the network should generate the sequence (*S, A, B, C*) in response to the input sequence (*s, a, b, b*). Then, the network learns a new sequence $\left(\xi_{1}^{2},\xi_{2}^{2},\xi_{3}^{2}\right)=\left(S,A^{\prime },B\right)$, which is associated with $\left(\eta_{1}^{2},\eta_{2}^{2},\eta_{3}^{2}\right)=\left(s,a^{\prime },b\right)$. In this study, we intend to examine if the inference of *C* is achieved selectively from (*S, A*′, *B*) in the presence of *b*. For it, we need to prevent the tight and trivial association between input *b* and the sub-sequence (*B, C*). For this purpose, the network is also postulated to learn the association between $\eta_{1}^{3}=b$ and $\xi_{1}^{3}=D$ as a distractor. We explore if the network generates the sequence (*S, A*′, *B, C*) in response to the input sequence (*s, a*′, *b, b*) after learning the association between (*S, A*′, *B*) and (*s, a*′, *b*).

**Figure 4:**
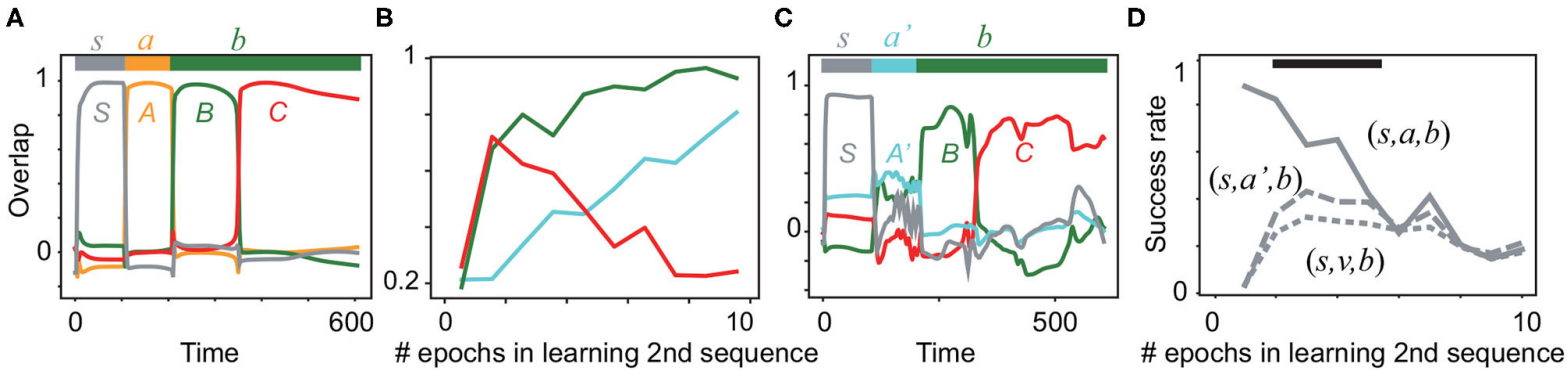
Neural activities in the inference task. **(A)** The neural dynamics in the recall of the first sequence (*S, A, B, C*) in response to (*s, a, b, b*) are plotted after the learning is completed. Color lines show the overlaps with *S*, *A*, *B*, and *C*. The bar at the top indicates the inputs (*s, a, b, b*) and their applied periods. **(B)** The average overlaps with the patterns *A*′, *B*, and *C* are plotted in cyan, green, and red, respectively, during the learning of the second sequence. Each overlap is obtained by averaging over 20 realizations of networks. **(C)** The neural dynamics in recall in response to (*s, a*′, *b, b*) are plotted by using the overlaps with *S*, *A*′, *B*, and *C* after three epochs of learning (*S, A*′, *B*). **(D)** Success rate of generation of the sub-sequence (*B, C*) is plotted for different input sequences (*s, a, b, b*), (*s, a*′, *b, b*), and (*s, v, b, b*) as a function of the learning epoch for the second sequence. The black bar indicates the region in which the success rates between (*s, a*′, *b, b*) and (*s, v, b, b*) are significantly different (Wilcoxon's signed-rank test *p* < 0.05). Refer to Materials and methods for details.

During the learning of the first sequence, the overlaps with all of the targets reach more than 0.9 after 20 epochs of learning, as shown in Supplementary Figure S4A. Actually, Figure 4A shows that the first sequence is successfully generated. Next, the network learns the new sequential patterns (*S, A*′, *B*). If the network infers (*S, A*′, *B, C*) by using the already learned sub-sequence (*B, C*), it generates the sequence (*S, A*′, *B, C*) after learning only (*S, A*′, *B*) without (*S, A*′, *B, C*). As expected, the average overlaps with all of the targets in the second sequence (not only *A*′,*B*,but also *C*) are increased through learning (Figure 4B). After the fifth epoch of learning, the overlap with *C* declines, whereas those with *A*′ and *B* continuously increase. As an example, we plot the recall dynamics after learning *A*′ and *B* in Figure 4C. *A*′ evokes *B* and *C*, although the overlap with the first target *A*′ is not large. Simultaneously, the network generates the first sequence (Supplementary Figure S4C). Note that the sub-sequence (*B, C*) is generated selectively by the input either (*s, a, b, b*) or (*s, a*′, *b, b*). In contrast, when a random input *v* is given instead of *a* or *a*′ in an input sequence, the sub-sequence (*B, C*)is not evoked, as shown in Supplementary Figure S4B. To examine the difference among the recall dynamics in response to (*s, a, b*), (*s, a*′, *b*), and (*s, v, b*), the success rate of generating the sub-sequence (refer to its definition in Materials and methods) is analyzed statistically in Figure 4D. We found that the input sequences (*s, a, b*), (*s, a*′, *b*) evoke the sub-sequence (*B, C*) with a significantly higher rate than the input sequence (*s, v, b*). Thus, our model is able to infer a new sequence based on the previously learned sequence.

### 3.3. Learning of History-Dependent Sequences

**Figure 5:**
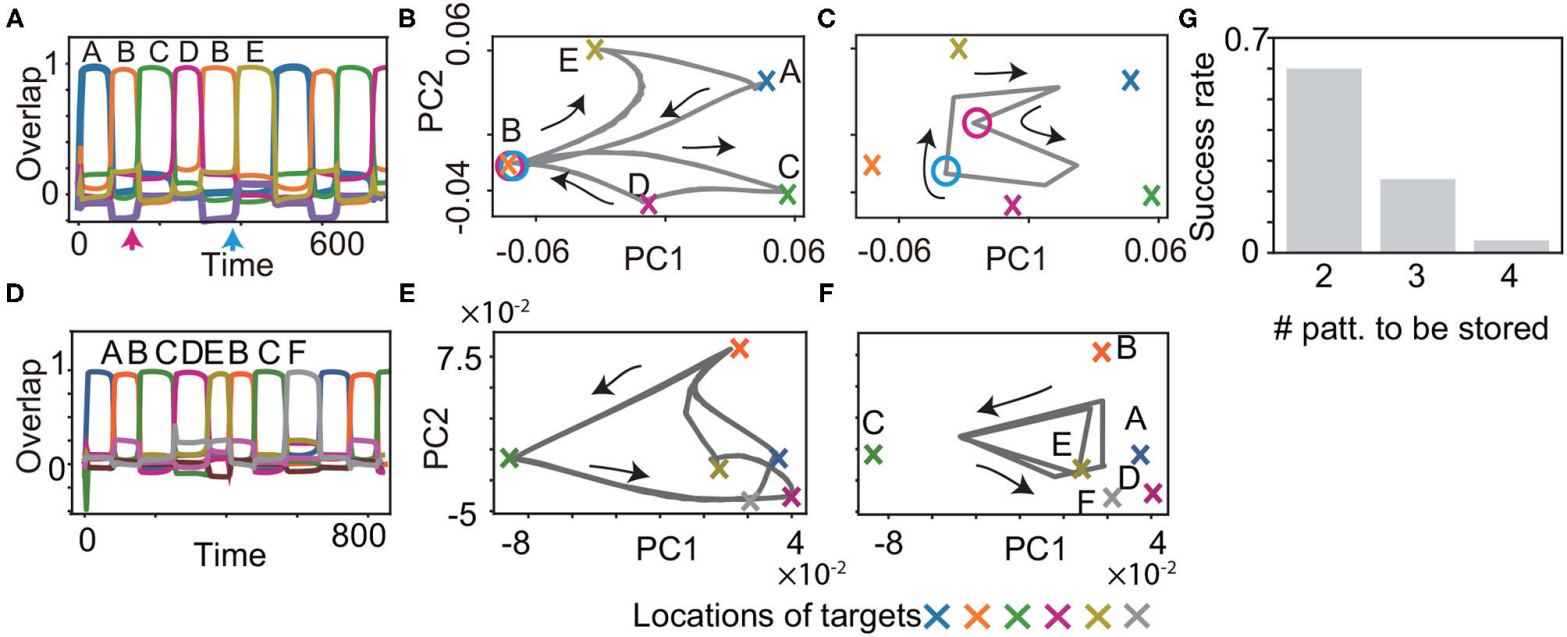
Recall processes for history-dependent sequences for *K* = 1, *M* = 6 **(A–C)** and for *K* = 1, *M* = 8 **(D–F)**. **(A** and **D)** The neural activities of ***x*** upon ***η***^1^ are plotted by using their overlaps with the targets. Colors and alphabets indicate which targets overlapped. **(B,C,E** and **F)** The neural dynamics plotted in **(A** and **D)** are shown by projecting the fast dynamics in **(B** and **E)** and the slow dynamics in **(C** and **F)** onto a 2-dimensional PC space. X-shaped marks represent the locations of the targets. Magenta and cyan circles in B indicate the locations of ***x***, respectively, just before targets *C* and *E* are recalled (as indicated by the arrows in **(A)**, whereas the circles in **(C)** indicate the locations of ***y***. **(G)** The success rate in generating these sequences is shown for different lengths. For generating the sequence with *M*, the network is required to store the information about *M*/2−1 preceding patterns. We measured the success rate over 50 network realizations and plotted it as a function of the number of the preceding patterns to be stored.

We examined if the proposed model learns the history-dependent sequence (*M* = 6), in which the same patterns exist in a sequence such as $\left(\xi_{1}^{1},\xi_{2}^{1},\cdots \;,\xi_{6}^{1}\right)=\left(A,B,C,D,B,E\right)$. The patterns succeeding *B* are *C* or *E*, depending on whether the previous pattern is *A* or *D*. Then, the neural dynamics have to retain the information of the target *A* or *D*, to recall the target *C* or *E* correctly. Our model succeeded in recalling this sequence, as shown in Figure 5A. Just before the target *C* and *E* are recalled, there is no clear difference in the values of fast variables ***x***, as indicated by the circles in Figure 5B. However, the values of slow variables ***y*** are different, depending on the previous targets shown in Figure 5C, which stabilize different patterns of ***x***. Furthermore, we demonstrate that our model succeeded in recalling more complex sequences (*M* = 8) such as $\left(\xi_{1}^{1},\xi_{2}^{1},\cdots \;,\xi_{8}^{1}\right)=\left(A,B,C,D,E,B,C,F\right)$, as shown in Figures 5D–F. In this case, the neural dynamics have to keep three previous targets in memory to recall the target *D* or *F* after *B* and *C*. As expected, generating the sequence with *M* = 8 is a harder task than that with *M* = 6. However, some networks still can generate the sequence.

To understand the performance comprehensively, we measured the success rate in generating these sequences for different lengths. For the sequence with the length of *M*, the network is required to retain the information about *M*/2−1 preceding patterns. We examined the success rate for *M* = 6, 8, and 10 over 50 networks realizations in the same manner as that in the inference task. The success rate is reduced for the longer *M* and nearly zero for *M* = 10. This result indicates that our model can store three preceding patterns at a maximum, but is difficult to memorize four preceding patterns.

**Figure 6:**
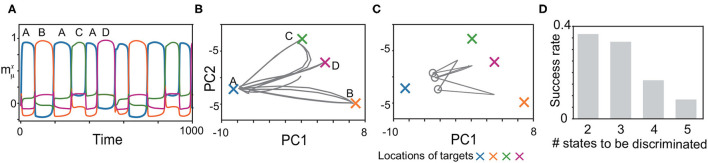
Recall processes for history-dependent sequences such as (*A, B, A, C, A, D*). **(A)** The neural activities of ***x*** upon ***η***^1^ are plotted by using their overlaps with the targets. **(B,C)** The neural dynamics plotted in **(A)** are shown by projecting the fast dynamics in **(B)** and the slow dynamics in **(C)** onto a 2-dimensional PC space. X-shaped marks represent the locations of the targets. Gray circles in **(C)** indicate the locations of ***y*** just before targets *B*, *C*, and *D* are recalled. **(D)** The success rate in generating these sequences is shown for different lengths. For generating the sequence with *M*, the network is required to discriminate *M*/2 neural states just before targets *B, C, D*, … are recalled. We measured the success rate over 50 network realizations and plotted it as a function of the number of the states to be discriminated.

We, next, explored whether the model can memorize another type of the history-dependent sequence such as $\left(\xi_{1}^{1},\xi_{2}^{1},\cdots \;,\xi_{6}^{1}\right)=\left(A,B,A,C,A,D\right)$, as shown in Figure 6. The network is required to discriminate three neural states in the slow dynamics just before ***x*** approaches *B*, *C*, and *D*, as shown by circles in Figure 6C. When the network discriminates these states successfully, it generates the sequence adequately, as shown in Figures 6A–C. We measured the success rate in generating these sequences for different numbers of the states to be discriminated (namely, *M*/2 states for an *M*-pattern sequence) in Figure 6D. For the shortest case, the success rate takes less than 0.4.

**Figure 7:**
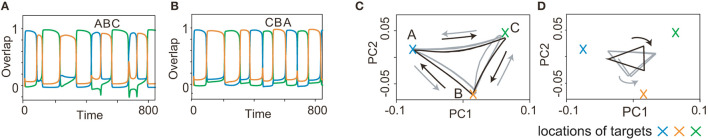
Recall processes for history-dependent sequences for *K* = 2, *M* = 3. **(A** and **B)** The neural activities of ***x*** upon ***η***^1^ and ***η***^2^ are plotted by using their overlaps with the targets in **(A** and **B)**, respectively. Colors and alphabets indicate the targets overlapped. **(C** and **D)** The neural dynamics shown in **(A** and **B)** are shown by projecting them onto a 2-dimensional PC space. The fast dynamics are shown in **(C)** and the slow dynamics are shown in **(D)**. The neural trajectories upon ***η***^1^ and ***η***^2^ are plotted in gray and black, respectively.

As a final example of the complex sequences, we explored learning two history-dependent sequences (Figure 7), namely, ($\xi_{1}^{1},\xi_{2}^{1},\xi_{3}^{1}$)=(*A, B, C*) upon ***η***^1^, and ($\xi_{1}^{2},\xi_{2}^{2},\xi_{3}^{2}$)=(*C, B, A*) upon ***η***^2^. In these sequences, the flow *A*→*B*→*C* on the state space under ***η***^1^ should be reversed under ***η***^2^. The learned network succeeds in generating these sequences. Although orbits of ***x*** under inputs almost overlap in the 2-dimensional space, those of ***y*** does not. This difference in ***y***, in addition to inputs, allows the orbits of ***x*** in the reverse order of patterns. Generally, ***y*** is different depending on the history of the previous patterns and inputs even when ***x*** is the same. Different ***y*** stabilizes different fixed point of ***x***, to generate the history-dependent sequence.

Generating these sequences is rather hard. Actually, the success rate is 0.14 for three-pattern sequences [(*A, B, C*) under one input, (*C, B, A*) under the other input] and 0.08 for four-pattern sequences. The success rate is low even for the shortest sequences. The difficulty of this task could be attributed to the request that networks have to memorize the bidirectional transitions between the target patterns (namely, the transition from *A* to *B* and its reverse transition) dependent on the external input.

### 3.4. Timescale Dependence

**Figure 8:**
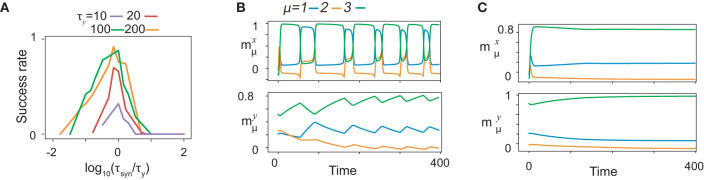
Time scale dependence of neural dynamics. **(A)** The success rate of recalls as functions of τ_*syn*_ for given τ_*y*_. The curves of the success rates are rescaled by τ_*y*_. Different colors represent different τ_*y*_ indicated by bars below panels. The success rate is calculated across fifty realizations for *K* = 1, *M* = 7. **(B** and **C)** The neural activities of ***x*** (upper) and ***y*** (lower) in the recall process are shown by using the overlaps with the targets in the same color as shown in Figure 2A. The neural dynamics for τ_*y*_ = 100, τ_*syn*_ = 10 are shown in B, while those for τ_*y*_ = 100, τ_*syn*_ = 1, 000 are shown in **(C)**.

Recall performance is highly dependent on the relation between τ_*x*_, τ_*y*_, and τ_*syn*_. To investigate the dependence of the performance on the timescales, we trained fifty realizations of networks for various values of timescales and calculated the success rate of training as a function of τ_*syn*_ for different τ_*y*_ by fixing τ_*x*_ at 1, as are plotted after rescaling τ_*syn*_ by τ_*y*_ in Figure 8A. The ratios yield a common curve that shows an optimal value ~1 at τ_*syn*_, approximately equal to τ_*y*_. As an exceptional case, the success rate for τ_*y*_ = 10 yields a lower value for the optimal τ_*syn*_ because τ_*y*_ is too close to τ_*x*_ to store the information about ***x***. The balance between τ_*syn*_ and τ_*y*_ is important to regulate the success rate when they are sufficiently smaller than τ_*x*_.

To unveil the significance of the timescale balance, we, first, present how the recall is failed for τ_*y*_>>τ_*syn*_, (τ_*y*_ = 100, τ_*syn*_ = 10 in Figure 8B). Some of the targets are recalled sequentially in the wrong order, whereas other targets do not appear in the recall process. To uncover the underlying mechanism of the failed recall, we analyze the neural dynamics of fast variables with slow variables quenched in a manner similar to that shown in Figure 2 (refer to Supplementary Figure S5A). Here, all the targets are stable for certain ***y***, although $\xi_{2}^{1}$ does not appear in the recall process. We also found that fixed points corresponding to $\xi_{1}^{1}$ and $\xi_{2}^{1}$ do not coexist for any ***y***: the fixed point corresponding to $\xi_{3}^{1}$ has a large basin across all ***y***. This leads to a transition from $\xi_{1}^{1}$ to $\xi_{3}^{1}$ by skipping $\xi_{2}^{1}$, and thus, the recall is failed.

Interestingly, how a recall is failed for τ_*y*_ < < τ_*syn*_ are distinct from that for τ_*y*_>>τ_*syn*_. For τ_*y*_ = 100, τ_*syn*_ = 1, 000, only the most recently learned target is stable for almost all ***y***, and thus, only this target is recalled, as shown in Figure 8C. We sampled the slow variables from the last learning step of the sequence (Supplementary Figure S5B), and analyzed the bifurcation of the fast variables against change in slow variables, in the same way as above. In this study, only the latest target (here, $\xi_{3}^{1}$) is a fixed point, whereas the other targets are not. Thus, transitions between targets are missed, except the transition to the latest target.

Why does the network fail in generating the sequences when τ_*y*_ and τ_*syn*_ are not matched? The reason is as follows: the learning time for the non-optimal timescales takes a longer time than that for the optimal ones. In this study, we consider the time that is normalized by τ_*syn*_ because it characterizes the timescale regulating the synaptic plasticity and, consequently, the neural dynamics. For τ_*y*_>>τ_*syn*_ (Supplementary Figure S5C), the trajectories of neural activities are similar to those for τ_*y*_~τ_*syn*_, but it takes a longer time for ***y*** to approach ***x*** after ***x*** converges to the target. As the approach to ***x*** is so slow, the learning process stabilizes the present target during the approach by modifying the connectivity, so that the target is too stable over a wide range of ***y***. On the other hand, for τ_*y*_ < < τ_*syn*_ (Supplementary Figure S5E), the neural activities of ***x*** and ***y*** wander before ***x*** converges to the target, resulting in disruption of information about the previous targets stored in ***y***. Thus, the networks for both cases of τ_*y*_>>τ_*syn*_ and τ_*y*_ < < τ_*syn*_ fail to generate the sequence. These results indicate that the relative timescale τ_*y*_ to τ_*syn*_ changes the bifurcation of the fast dynamics and the memory capacity.

## 4. Discussion

Sequential transitions between metastable patterns are ubiquitously observed in the neural system (Miller, 2016) during various tasks, such as perception (Jones et al., 2007; Miller and Katz, 2010), decision making (Ponce-Alvarez et al., 2012), working memory (Stokes et al., 2013; Taghia et al., 2018),and recall of long-term memory (Wimmer et al., 2020). We have developed a novel neural network model with fast and slow dynamics to generate sequences with non-Markov property and concatenate sequences, which are based on these cognitive functions.

In a standard model for generating sequential patterns (Kleinfeld, 1986; Sompolinsky and Kanter, 1986; Nishimori et al., 1990; Russo and Treves, 2012; Recanatesi et al., 2015; Haga and Fukai, 2019), asymmetric Hebbian learning between a pattern μ and the next μ+1, i.e., **ξ***^μ+1^*(**ξ***^μ^*)*t*, is used to create the transition from **ξ***^μ^* to **ξ**^μ+1^ (Kleinfeld, 1986; Sompolinsky and Kanter, 1986; Russo and Treves, 2012; Recanatesi et al., 2015; Haga and Fukai, 2019). In these studies, however, only the connections between the current and immediately preceding patterns are embedded in the connectivity, resulting in that the prolonged history of the patterns cannot be embedded. In other studies, Rabinovich's group (Seliger et al., 2003; Rabinovich and Varona, 2018) proposed the model generating sequential activities by heteroclinic orbits between patterns. As the above standard model, asymmetric Hebbian learning forms the connectivity for generating the sequences (Seliger et al., 2003). The information about the history of patterns is not stored in the model. Thus, non-Markov sequences are not generated in contrast to our model^3^.

In some models, a term that changes slower than the neural dynamics (e.g., an adaptation term) is introduced to lead to the transition. In Gros (2007); Russo and Treves (2012), and Recanatesi et al. (2015), the slow term is introduced to destabilize the current pattern. These methods imply non-Markov dynamics because the slow term needs prolonged times to recover, leading to change in the transition probabilities among the patterns. However, this term does not determine the next pattern and, thus, some additional mechanism is necessary for the transition to the desired pattern. The feedback from the slow population in our model, in contrast, not only destabilizes the current pattern but also simultaneously stabilizes the next targeted pattern. As the current and next patterns coexist for some time, the robust transition between them is achieved.

Alternatively, supervised learning methods used in machine learning fields, such as Back-Propagation Through Time (BPTT) (Werbos, 1990), are investigated to reproduce sequential neural activities observed experimentally (Mante et al., 2013; Carnevale et al., 2015; Chaisangmongkon et al., 2017), including non-Markov trajectories (Sussillo and Abbott, 2009; Laje and Buonomano, 2013). The BPTT, however, requires non-local information and the network has to retain a large amount of information until the trajectory terminates, which is biologically implausible. Furthermore, the trajectories shaped by this method are vulnerable to noise (Laje and Buonomano, 2013). Our model is free from these deficiencies.

In our model, the recurrent connections in the fast population (i.e., connections within a cortical area) are modified to shape the transitions between memorized states whereas the connections between the fast and slow populations and those from the input to the fast population are fixed (i.e., connections across cortical areas). In another approach, Gros and Kaczor (2010) demonstrated that the plasticity in the afferent connections with the fixed recurrent connections is useful for semantic learning, by connecting appropriately external stimuli with already established neural patterns in the recurrent network. In the neural system, generally, both connections across and within cortical areas are plastic. The existence of both dual plasticity possibly leads to interference between these connections, potentially resulting in reducing the learning performance. Future studies are needed to clarify how such dual plasticity cooperatively builds neural activities to perform cognitive functions.

Timescales in the neural activities are hierarchically distributed across several cortical areas (Honey et al., 2012; Murray et al., 2014; Hasson et al., 2015; Runyan et al., 2017). For instance, consider the hippocampus (HPC) and the prefrontal cortex (PFC), which are coupled by mono-synaptic and di-synaptic connections (Ito et al., 2015). HPC neurons respond to the location of animals (Kumaran et al., 2016) with faster timescales than those in PFC, which has the slowest timescale among cortical areas (Murray et al., 2014). Experimental studies (Ito et al., 2015; Guise and Shapiro, 2017) revealed that PFC neurons are necessary to differentiate HPC dynamics depending on the context and previous experience. Similarly, neurons in the orbitofrontal cortex (OFC), whose timescales are considered to be slower than those in HPC, are necessary for concatenating the sequences in the stimulus-reward response (Jones et al., 2012; Wikenheiser and Schoenbaum, 2016). Accordingly, it is suggested that the area with the slow dynamics is necessary to generate and concatenate the sequences.

Neural networks with multiple timescales are investigated theoretically in several studies. In some studies (Yamashita and Tani, 2008; Perdikis et al., 2011), the slow dynamics are introduced to concatenate primitive movements and produce a complex movement, while hidden states of the hierarchical external stimuli are inferred by the multiple timescales in the neural dynamics in another study (Kiebel et al., 2009). In Kiebel et al. (2009) and Perdikis et al. (2011), the relationship between the slow and fast dynamics are fixed a priori to perform their tasks, whereas, in our model, such a relationship is shaped through the learning process. In Yamashita and Tani (2008), the BPTT method is adopted for training the network; thus, it faced the same drawbacks as already mentioned. In the studies of the multiple timescales system, analytical methods such that singular perturbation methods are adopted, which are commonly used to elucidate the transition between the states on the different slow manifolds (Ermentrout, 1998; Rubin et al., 2013; Bertram and Rubin, 2017; Wernecke et al., 2018) and the stability of fixed points (Meyer-Bäse et al., 1996; Hongtao and Amari, 2006). Our model provides how these transitions are formed through learning and it generates and concatenates the history-dependent sequences, while the application of these methods will be useful in the future.

As for the timescales, we need further studies to fill a gap between our model and experimental observations. The ratio of the timescale in the slow dynamics to that in the fast dynamics is less than 10 times across cortical areas (Wang and Kennedy, 2016), which are smaller than the optimal ratio in our model. Further, the difference between the timescales in the slow dynamics (on the order of a second) and in the synaptic plasticity (on the order of a minute Bliss and Lomo, 1973; Bayazitov et al., 2007) is larger than that adopted in our model.

Diversity in the timescales of individual neurons and the calcium dynamics possibly resolve this discrepancy. The timescale of individual neurons in the same area is distributed over two digits (Bernacchia et al., 2011; Wasmuht et al., 2018). The calcium dynamics in the synapses can modify the synaptic efficacy on the order of a second (Shouval et al., 2010; Graupner and Brunel, 2012). By taking these effects into account, our model may be consistent with the experimental observations, although further studies will be important, including those with spiking neurons (Kurikawa and Kaneko, 2015) and spike-timing-dependent potentiation.

Finally, we discuss the biological plausibility of the learning rule in our model. The fast network receives two inputs; an external input (**η**) and the input from the slow network. In the neural system, the external input is conveyed through afferent connections from a lower cortical area (or sensory input) and the feedback input comes from a higher cortical area. In addition to these inputs, another input for the learning is introduced in our model, which provides information to generate sequential patterns to be learned. Thus, our network is trained to map between sensory cues and sequential patterns in the output area by using a Hebbian rule (correlation between ξ_*j*_ and *x*_*i*_) and an anti-Hebbian rule (correlation between *x*_*i*_ and *x*_*j*_). After training, the network evokes the sequential patterns under the sensory input.

## Data Availability Statement

The original contributions presented in the study are included in the article/Supplementary Material, further inquiries can be directed to the corresponding author.

## Author Contributions

TK and KK: conceptualization and writing. TK: formal analysis. Both authors contributed to the article and approved the submitted version.

## Funding

This study was partly supported by JSPS KAKENHI (Nos. 18K15343 and 20H00123).

## Conflict of Interest

The authors declare that the research was conducted in the absence of any commercial or financial relationships that could be construed as a potential conflict of interest.

## Publisher's Note

All claims expressed in this article are solely those of the authors and do not necessarily represent those of their affiliated organizations, or those of the publisher, the editors and the reviewers. Any product that may be evaluated in this article, or claim that may be made by its manufacturer, is not guaranteed or endorsed by the publisher.

## Footnotes

^1^According to Eq. (4), $d\left(\underset{j\neq i}{\sum }{\left(J_{ij}^{X}\right)}^{2}\right)/dt\propto \left(1-\underset{j\neq i}{\sum }{\left(J_{ij}^{X}\right)}^{2}\right)$.

^2^The other fixed point corresponding to $\xi_{3}^{1}$ also appears, but its basin is quite small. Thus, we can neglect this fixed point.

^3^It is shown that finely designed networks realize the history-dependent sequences (Chartier and Boukadoum, 2006; Verduzco-Flores et al., 2012). However, it is shown that the additional neurons or sub-networks as many as the number of memories or sequences are required in these studies.

## References

[B1] Akhlaghpour H. Wiskerke J. Choi J. Y. Taliaferro J. P. Au J. Witten I. B. (2016). Dissociated sequential activity and stimulus encoding in the dorsomedial striatum during spatial working memory. Elife 5, 1–20. 10.7554/eLife.19507 27636864 PMC5053805

[B2] Amari S.-I.. (1972). Learning patterns and pattern sequences by self-organizing nets of threshold elements. IEEE Trans. Comput. 100, 1197–1206. 10.1109/T-C.1972.22347727295638

[B3] Bayazitov I. T. Richardson R. J. Fricke R. G. Zakharenko S. S. (2007). Slow presynaptic and fast postsynaptic components of compound long-term potentiation. J. Neurosci. 27, 11510–11521. 10.1523/JNEUROSCI.3077-07.2007 17959794 PMC6673206

[B4] Bernacchia A. Seo H. Lee D. Wang X.-J. J. (2011). A reservoir of time constants for memory traces in cortical neurons. Nat. Neurosci. 14, 366–372. 10.1038/nn.2752 21317906 PMC3079398

[B5] Bertram R. Rubin J. E. (2017). Multi-timescale systems and fast-slow analysis. Math. Biosci. 287, 105–121. 10.1016/j.mbs.2016.07.003 27424950

[B6] Bliss T. V. Lomo T. (1973). Long-lasting potentiation of synaptic transmission in the dentate area of the anaesthetized rabbit following stimulation of the perforant path. J. Physiol. 232, 331–356. 10.1113/jphysiol.1973.sp010273 4727084 PMC1350458

[B7] Carnevale F. DeLafuente V. Romo R. Barak O. Parga N. de Lafuente V.. (2015). Dynamic control of response criterion in premotor cortex during perceptual detection under temporal uncertainty. Neuron 86, 1067–1077. 10.1016/j.neuron.2015.04.014 25959731

[B8] Chaisangmongkon W. Swaminathan S. K. Freedman D. J. Wang X.-J. J. (2017). Computing by robust transience: how the fronto-parietal network performs sequential, category-based decisions. Neuron 93, 1504.e4–1517.e4. 10.1016/j.neuron.2017.03.002 28334612 PMC5586485

[B9] Chartier S. Boukadoum M. (2006). A sequential dynamic heteroassociative memory for multistep pattern recognition and one-to-many association. IEEE Trans. Neural Netw. 17, 59–68. 10.1109/TNN.2005.860855 16526476

[B10] Chaudhuri R. Knoblauch K. Gariel M. A. Kennedy H. Wang X. J. (2015). A large-scale circuit mechanism for hierarchical dynamical processing in the primate cortex. Neuron 88, 419–431. 10.1016/j.neuron.2015.09.008 26439530 PMC4630024

[B11] Ermentrout B.. (1998). Neural networks as spatio-temporal pattern-forming systems. Rep. Progr. Phys. 61, 353–430. 10.1088/0034-4885/61/4/002

[B12] Graupner M. Brunel N. (2012). Calcium-based plasticity model explains sensitivity of synaptic changes to spike pattern, rate, and dendritic location. Proc. Natl. Acad. Sci. U.S.A. 109, 21551–21551. 10.1073/pnas.1109359109 22357758 PMC3309784

[B13] Gros C.. (2007). Neural networks with transient state dynamics. New J. Phys. 9:109. 10.1088/1367-2630/9/4/109

[B14] Gros C. Kaczor G. (2010). Semantic learning in autonomously active recurrent neural networks. Logic J. IGPL 18, 686–704. 10.1093/jigpal/jzp045

[B15] Guise K. G. Shapiro M. L. (2017). Medial prefrontal cortex reduces memory interference by modifying hippocampal encoding. Neuron. 94, 183.e8–192.e8. 10.1016/j.neuron.2017.03.011 28343868 PMC5398284

[B16] Gupta A. S. van der Meer M. A. Touretzky D. S. Redish A. D. (2010). hippocampal replay is not a simple function of experience. Neuron 65, 695–705. 10.1016/j.neuron.2010.01.034 20223204 PMC4460981

[B17] Haga T. Fukai T. (2019). Extended temporal association memory by modulations of inhibitory circuits. Phys. Rev. Lett. 123, 78101. 10.1103/PhysRevLett.123.078101 31491118

[B18] Hasson U. Chen J. Honey C. J. (2015). Hierarchical process memory: memory as an integral component of information processing. Trends Cogn. Sci. 19, 304–313. 10.1016/j.tics.2015.04.006 25980649 PMC4457571

[B19] Honey C. J. Thesen T. Donner T. H. Silbert L. J. Carlson C. E. Devinsky O.. (2012). Slow cortical dynamics and the accumulation of information over long timescales. Neuron 76, 423–434. 10.1016/j.neuron.2012.08.011 23083743 PMC3517908

[B20] Hongtao L. Amari S. I. (2006). Global exponential stability of multitime scale competitive neural networks with nonsmooth functions. IEEE Trans. Neural Netw. 17, 1152–1164. 10.1109/TNN.2006.875995 17001977

[B21] Ito H. T. Zhang S.-,j. Witter M. P. Moser E. I. Moser M.-B. (2015). A prefrontal-thalamo-hippocampal circuit for goal-directed spatial navigation. Nature 522, 50–55. 10.1038/nature14396 26017312

[B22] Jin X. Tecuapetla F. Costa R. M. (2014). Basal ganglia subcircuits distinctively encode the parsing and concatenation of action sequences. Nat. Neurosci. 17, 423–430. 10.1038/nn.3632 24464039 PMC3955116

[B23] Jones J. L. Esber G. R. McDannald M. A. Gruber A. J. Hernandez A. Mirenzi A.. (2012). Orbitofrontal cortex supports behavior and learning using inferred but not cached values. Science 338, 953–956. 10.1126/science.1227489 23162000 PMC3592380

[B24] Jones L. M. Fontanini A. Sadacca B. F. Miller P. Katz D. B. (2007). Natural stimuli evoke dynamic sequences of states in sensory cortical ensembles. Proc. Natl. Acad. Sci. U.S.A. 104, 18772–18777. 10.1073/pnas.0705546104 18000059 PMC2141852

[B25] Kiebel S. J. Daunizeau J. Friston K. J. (2008). A hierarchy of time-scales and the brain. PLoS Comput. Biol. 4:e1000209. 10.1371/journal.pcbi.1000209 19008936 PMC2568860

[B26] Kiebel S. J. von Kriegstein K. Daunizeau J. Friston K. J. (2009). Recognizing sequences of sequences. PLoS Comput. Biol. 5:e1000464. 10.1371/journal.pcbi.1000464 19680429 PMC2714976

[B27] Kleinfeld D.. (1986). Sequential state generation by model neural networks. Proc. Natl. Acad. Sci. U.S.A. 83, 9469–9473. 10.1073/pnas.83.24.9469 3467316 PMC387161

[B28] Kumaran D. Hassabis D. McClelland J. L. (2016). What learning systems do intelligent agents need? complementary learning systems theory updated. Trends Cogn. Sci 20, 512–534. 10.1016/j.tics.2016.05.004 27315762

[B29] Kurikawa T. Barak O. Kaneko K. (2020). Repeated sequential learning increases memory capacity via effective decorrelation in a recurrent neural network. Phys. Rev. Res. 2, 023307. 10.1103/PhysRevResearch.2.023307

[B30] Kurikawa T. Haga T. Handa T. Harukuni R. Fukai T. (2018). Neuronal stability in medial frontal cortex sets individual variability in decision-making. Nat. Neurosci. 21, 1764–1773. 10.1038/s41593-018-0263-5 30420732

[B31] Kurikawa T. Kaneko K. (2013). Embedding responses in spontaneous neural activity shaped through sequential learning. PLoS Comput. Biol. 9:e1002943. 10.1371/journal.pcbi.1002943 23505355 PMC3591288

[B32] Kurikawa T. Kaneko K. (2015). Memories as bifurcations: Realization by collective dynamics of spiking neurons under stochastic inputs. Neural Netw. 62, 25–31. 10.1016/j.neunet.2014.07.005 25124069

[B33] Kurikawa T. Kaneko K. (2016). Dynamic organization of hierarchical memories. PLoS ONE 11:e0162640. 10.1371/journal.pone.0162640 27618549 PMC5019405

[B34] Laje R. Buonomano D. V. (2013). Robust timing and motor patterns by taming chaos in recurrent neural networks. Nat. Neurosci. 16, 925–933. 10.1038/nn.3405 23708144 PMC3753043

[B35] Larkum M.. (2013). A cellular mechanism for cortical associations: an organizing principle for the cerebral cortex. Trends Neurosci. 36, 141–151. 10.1016/j.tins.2012.11.006 23273272

[B36] Larkum M. E. Nevian T. Sandler M. Polsky A. Schiller J. (2009). Synaptic integration in tuft dendrites of layer 5 pyramidal neurons: a new unifying principle. Science 325, 756–760. 10.1126/science.1171958 19661433

[B37] Maboudi K. Ackermann E. de Jong L. W. Pfeiffer B. E. Foster D. Diba K.. (2018). Uncovering temporal structure in hippocampal output patterns. Elife 7, 1–24. 10.7554/eLife.34467 29869611 PMC6013258

[B38] Mante V. Sussillo D. Shenoy K. V. Newsome W. T. (2013). Context-dependent computation by recurrent dynamics in prefrontal cortex. Nature 503, 78–84. 10.1038/nature12742 24201281 PMC4121670

[B39] Mazzucato L. Fontanini A. La Camera G. (2015). Dynamics of multistable states during ongoing and evoked cortical activity. J. Neurosci. 35, 8214–8231. 10.1523/JNEUROSCI.4819-14.2015 26019337 PMC4444543

[B40] Meyer-Bäse A. Ohl F. Scheich H. (1996). Singular perturbation analysis of competitive neural networks with different time scales. Neural Comput. 8, 1731–1742. 10.1162/neco.1996.8.8.1731 8888615

[B41] Miller P.. (2016). Itinerancy between attractor states in neural systems. Curr. Opin Neurobiol. 40, 14–22. 10.1016/j.conb.2016.05.005 27318972 PMC5056802

[B42] Miller P. Katz D. B. (2010). Stochastic transitions between neural states in taste processing and decision-making. J. Neurosci. 30, 2559–2570. 10.1523/JNEUROSCI.3047-09.2010 20164341 PMC2851230

[B43] Murray J. D. Bernacchia A. Freedman D. J. Romo R. Wallis J. D. Cai X.. (2014). A hierarchy of intrinsic timescales across primate cortex. Nat. Neurosci. 17, 1661–1663. 10.1038/nn.3862 25383900 PMC4241138

[B44] Nishimori H. Nakamura T. Shiino M. (1990). Retrieval of spatio-temporal sequence in asynchronous neural network. Phys. Rev. A 41, 3346–3354. 10.1103/PhysRevA.41.3346 9903491

[B45] Perdikis D. Huys R. Jirsa V. K. (2011). Time scale hierarchies in the functional organization of complex behaviors. PLoS Comput. Biol. 7:e1002198. 10.1371/journal.pcbi.1002198 21980278 PMC3182871

[B46] Ponce-Alvarez A. Nácher V. Luna R. Riehle A. Romo R. (2012). Dynamics of cortical neuronal ensembles transit from decision making to storage for later report. J. Neurosci. 32, 11956–11969. 10.1523/JNEUROSCI.6176-11.2012 22933781 PMC3865507

[B47] Rabinovich M. I. Varona P. (2018). Discrete sequential information coding: heteroclinic cognitive dynamics. Front. Comput. Neurosci. 12:73. 10.3389/fncom.2018.00073 30245621 PMC6137616

[B48] Recanatesi S. Katkov M. Romani S. Tsodyks M. (2015). Neural network model of memory retrieval. Front. Comput. Neurosci. 9:149. 10.3389/fncom.2015.00149 26732491 PMC4681782

[B49] Rubin J. J. Rubin J. E. Ermentrout G. B. (2013). Analysis of synchronization in a slowly changing environment: how slow coupling becomes fast weak coupling. Phys. Rev. Lett. 110, 204101. 10.1103/PhysRevLett.110.204101 25167415

[B50] Runyan C. A. Piasini E. Panzeri S. Harvey C. D. (2017). Distinct timescales of population coding across cortex. Nature 548, 92–96. 10.1038/nature23020 28723889 PMC5859334

[B51] Russo E. Treves A. (2012). Cortical free-association dynamics: distinct phases of a latching network. Phys. Rev. E 85, 1–21. 10.1103/PhysRevE.85.051920 23004800

[B52] Schuck N. W. Niv Y. (2019). Sequential replay of nonspatial task states in the human hippocampus. Science 364. 10.1126/science.aaw5181 31249030 PMC7241311

[B53] Seliger P. Tsimring L. S. Rabinovich M. I. (2003). Dynamics-based sequential memory: winnerless competition of patterns. Phys. Rev. E 67, 4. 10.1103/PhysRevE.67.011905 12636530

[B54] Shouval H. Z. Wang S. S. Wittenberg G. M. (2010). Spike timing dependent plasticity: a consequence of more fundamental learning rules. Front. Comput. Neurosci. 4:19. 10.3389/fncom.2010.00019 20725599 PMC2922937

[B55] Sompolinsky H. Kanter I. (1986). Temporal association in asymmetric neural networks. Phys. Rev. Lett. 57, 2861–2864. 10.1103/PhysRevLett.57.2861 10033885

[B56] Stokes M. G. Kusunoki M. Sigala N. Nili H. Gaffan D. Duncan J. (2013). Dynamic coding for cognitive control in prefrontal cortex. Neuron 78, 364–375. 10.1016/j.neuron.2013.01.039 23562541 PMC3898895

[B57] Sussillo D. Abbott L. F. (2009). Generating coherent patterns of activity from chaotic neural networks. Neuron 63, 544–557. 10.1016/j.neuron.2009.07.018 19709635 PMC2756108

[B58] Taghia J. Cai W. Ryali S. Kochalka J. Nicholas J. Chen T.. (2018). Uncovering hidden brain state dynamics that regulate performance and decision-making during cognition. Nat. Commun. 9. 10.1038/s41467-018-04723-6 29950686 PMC6021386

[B59] Verduzco-Flores S. O. Bodner M. Ermentrout B. Oscar S. Bodner V.-F. M. (2012). A model for complex sequence learning and reproduction in neural populations. J. Comput. Neurosci. 32, 403–423. 10.1007/s10827-011-0360-x 21887499

[B60] Wang X.-,j. Kennedy H (2016). Brain structure and dynamics across scales : in search of rules. Curr. Opin. Neurobiol. 37, 92–98. 10.1016/j.conb.2015.12.010 26868043 PMC5029120

[B61] Wasmuht D. F. Spaak E. Buschman T. J. Miller E. K. Stokes M. G. (2018). Intrinsic neuronal dynamics predict distinct functional roles during working memory. Nat. Commun. 9, 3499. 10.1038/s41467-018-05961-4 30158572 PMC6115413

[B62] Werbos P. J.. (1990). Backpropagation through time: what it does and how to do it. Proc. IEEE 78, 1550–1560. 10.1109/5.5833727295638

[B63] Wernecke H. Sándor B. Gros C. (2018). Attractor metadynamics in terms of target points in slow-fast systems: adiabatic versus symmetry protected flow in a recurrent neural network. J. Phys. Commun. 2, 095008. 10.1088/2399-6528/aac33c

[B64] Wikenheiser A. M. Schoenbaum G. (2016). Over the river, through the woods: cognitive maps in the hippocampus and orbitofrontal cortex. Nat. Rev. Neurosci. 17, 513–523. 10.1038/nrn.2016.56 27256552 PMC5541258

[B65] Wimmer G. E. Liu Y. Vehar N. Behrens T. E. Dolan R. J. (2020). Episodic memory retrieval success is associated with rapid replay of episode content. Nat. Neurosci. 23, 1025–1033. 10.1038/s41593-020-0649-z 32514135 PMC7610376

[B66] Yamashita Y. Tani J. (2008). Emergence of functional hierarchy in a multiple timescale neural network model: a humanoid robot experiment. PLoS Comput. Biol. 4:e1000220. 10.1371/journal.pcbi.1000220 18989398 PMC2570613

