# Supplementary material for: Multiple-Timescale Neural Networks: Generation of History-Dependent Sequences and Inference Through Autonomous Bifurcations
Source: Front Comput Neurosci. 2021 Dec 10;15:743537. doi: 10.3389/fncom.2021.743537 (PMC8702558; doi:10.3389/fncom.2021.743537)
Supplement: Supplementary file 1 [file Data_Sheet_1.pdf]

Supplemental materials of "Multiple-timescale Neural Networks: Generation of History-dependent Sequences and Inference through Autonomous Bifurcations"

Tomoki Kurikawa[1,*], Kunihiko Kaneko[2,3]

1 Department of Physics, Kansai Medical University, Shinmachi 2-5-1, Hirakata, Osaka, Japan
2 Department of Basic science, Graduate school of arts and sciences, University of Tokyo, Komaba 3-8-1, Meguro-ku, Tokyo, Japan
3 Center for Complex Systems Biology, Universal Biology Institute, University of Tokyo, Komaba 3-8-1, Meguro-ku, Tokyo, Japan

*** kurikawa@hirakata.kmu.ac.jp**

1 Supplemental text

1.1 Dependence of the success rate of generating the sequences on the parameters

We examine how the parameters, β_x, β_y, and c affect the behavior in our model. To this end, we explore the success rate of generating the sequences without the history dependence for $K = 1, M = 5$.

Figure S1A shows the success rate for different β_x without changing other parameters. We found that the smaller β ($\beta < 1$) radically reduces the number of memories. On the other hand, the larger β does not reduce the number of memories. For larger β, however, even in the success trial, some of the sequence patterns intermittently disappear because of strongly chaotic dynamics that is hard to generate stable sequences. Thus, the stability in the recall process is not high for large β.

Figure S1B shows the success rate for different β_y without changing other parameters. For the intermediate value of β ($5 \leq \beta_y \leq 20$), the success rate is almost unity. In contrast, for much smaller or much larger β_y, the success rate is reduced.

Finally, figure S1C shows the success rate for different values of c without changing other parameters. $2\rho c^2/N$ equals the variance of J^{XY}, which controls the ratio of the strength of input from Y to X to that from the external input. For much smaller c (namely, $c = 1$), the success rate is drastically reduced. Although generating the sequence requires the information of previous patterns, the relatively small input from Y in which the information is stored cannot evoke the correct pattern in X.

1.2 Learning multiple sequences

Our model memorizes several sequences for different inputs. We exemplify the procedure of memorization by focusing on the learning and recall process for $K = 2$. Learning two sequences are accomplished in a manner similar to the learning of a single sequence, as described in the main text. The model learns two sequences alternatively: the first sequence $(\boldsymbol{\xi}_1^1, \boldsymbol{\xi}_1^1, \cdots, \boldsymbol{\xi}_M^1, \boldsymbol{\xi}_1^1)$ is learned with the same criteria for $K = 1$. After resetting the fast and slow variables, the second sequence $(\boldsymbol{\xi}_1^2, \boldsymbol{\xi}_2^2, \cdots, \boldsymbol{\xi}_M^2, \boldsymbol{\xi}_1^2)$ is learned in the same way. We repeated these processes 20 times before finishing the learning.

Fig. S2 shows a recall process for $K = 2, M = 3$ after learning. In the presence of $\boldsymbol{\eta}^{\mathbf{1}}$, the sequence $(\boldsymbol{\xi}_{\mathbf{1}}^{\mathbf{1}}, \boldsymbol{\xi}_{\mathbf{2}}^{\mathbf{1}}, \boldsymbol{\xi}_{\mathbf{3}}^{\mathbf{1}})$ is recalled, as shown in the figure. Then, after switching the input from $\boldsymbol{\eta}^{\mathbf{1}}$ to $\boldsymbol{\eta}^{\mathbf{2}}$ at $t = 1000$, the required sequence $(\boldsymbol{\xi}_{\mathbf{1}}^{\mathbf{2}}, \boldsymbol{\xi}_{\mathbf{2}}^{\mathbf{2}}, \boldsymbol{\xi}_{\mathbf{3}}^{\mathbf{2}})$ is recalled successfully.

1.3 Robustness of the sequences

We investigated the robustness of the sequence recall. First, we applied strong one-shot perturbations into the neural dynamics, where multiplicative noise was added to the neural activities of all neurons x_i and y_i, as $x_i \to (1 - r_i^x)x_i$, $y_i \to (1 - r_i^y)y_i$ $(i = 1, 2, \ldots, N)$, and $r_i^{x,y}$ was chosen randomly from a uniform distribution of 0 to 1. The trajectory with the one-shot perturbation is shown in Fig. S3B. After the perturbation, the neural dynamics rapidly recover to a limit cycle, in which the neural activity exhibits a transition from one target to another in the correct order.

Next, we examined the robustness against the change in the initial states and noise. Here, Gaussian white noise $\boldsymbol{\zeta}(t)$ was added into the neural dynamics $\boldsymbol{x}$ and $\boldsymbol{y}$ given by Eqs. (1,2) with satisfying $< \zeta_i(t)\zeta_j(t') >= s\delta_{ij}\delta(t - t')$ for $i = j$; otherwise 0. Here, δ_{ij} and δ are the Kronecker and Dirac delta, respectively, and s is the noise strength. Fig. S3A shows a trajectory from a random initial state under the noise by using the overlaps of the slow and fast variables for $K = 1, M = 5$. After the transient period, the trajectory converges to the limit cycle that generates the correct sequence recall. We tested nine other trajectories under noise from nine random conditions, and found that all trajectories converge to the limit cycle.

Furthermore, the robustness of the model against noise strength was examined. The dynamics of $\boldsymbol{x}$ for increasing the noise strength are plotted in Fig. S3C. Below $s = 0.3$, the sequence is recalled with the correct order. For stronger noise ($s = 0.5$), only a few patterns are recalled intermittently, and others are not. Fig. S3D(i) shows the success rate of recalls as a function of the noise strength. The success rate is approximately 0.8 (same as the ratio in the case without noise) up to $s = 0.1$, and decreases rapidly. All of these results demonstrate that the sequential patterns in our model are quite robust against changes in the initial states and noise.

Finally, we measured the duration for which the fast dynamics stay on each target. The duration measured with noise is normalized by that measured without noise. The normalized duration is plotted as a function of the noise strength in Fig. S3D(ii). We found that the normalized duration decreases as the noise strength increases. After the fast dynamics converge to the target attractor, the basin volume reduces over time, as shown in Fig. 2. Thus, stronger noise is likely to kick out the neural states from the targets earlier, resulting in a decrease in duration as the noise strength increases.

2 Supplemental figures

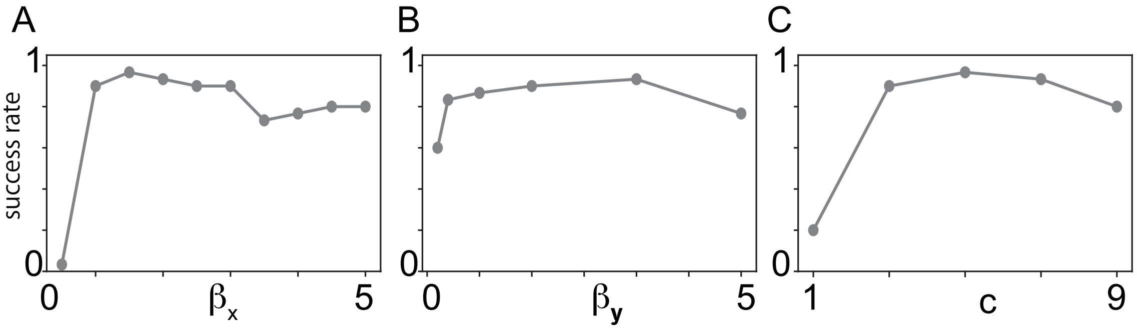

Fig.S 1. The success rates in generating the sequences are shown for $M = 5$. We measured the success rate for change in parameters over 30 network realizations. A: The success rate with change in β_x in Eq. 1 is plotted, while that with change in β_y in Eq. 2 and c are plotted in B and C, respectively.

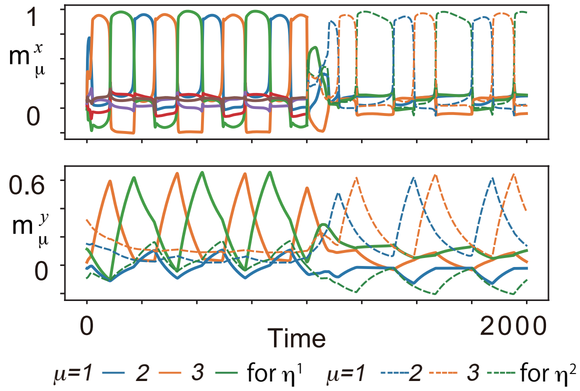

Fig.S 2. Recall dynamics for $K = 2, M = 3$ when the input is switched at $t = 1000$. The fast variables (top) and slow variables (bottom) are plotted by using the overlap m^{α}_{μ} ($\alpha = 1, 2$ and $\mu = 1, 2, 3$). The index of the overlap is indicated below the panels.

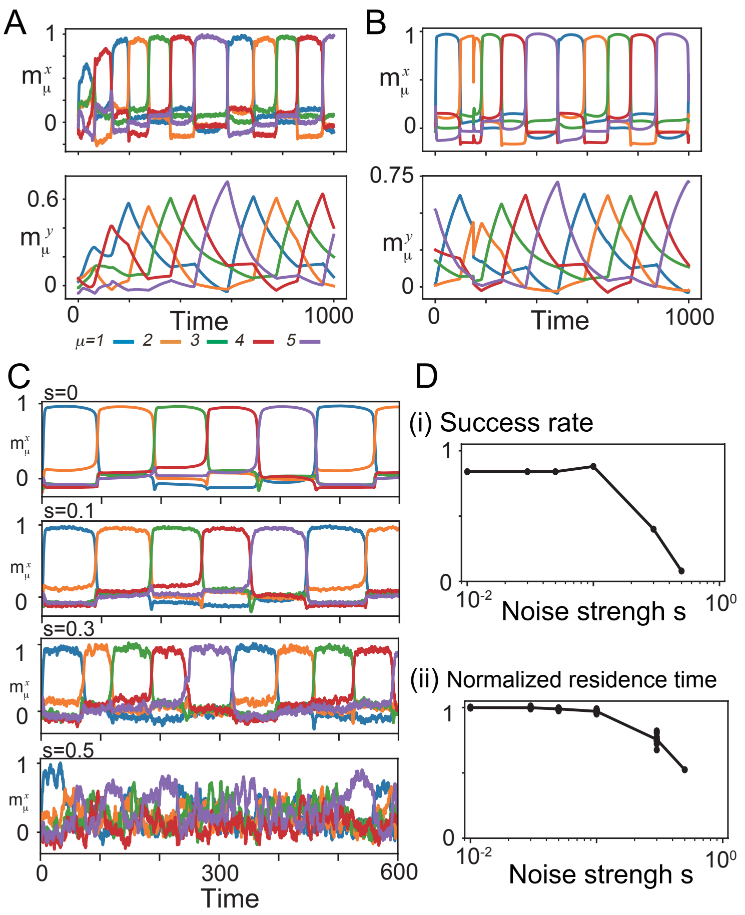

Fig.S 3. A and B: Trajectory of the overlaps of $\boldsymbol{x}$ (upper panel) and $\boldsymbol{y}$ (lower panel). The trajectory with the targets for noise strength $s = 0.1$ is shown in A, and that with one-shot perturbation (at $t = 150$) is shown in B. Each color indicates the target used in the calculation of the overlap (the same color code is used in the following panels). C: The time series of $\boldsymbol{x}$ are plotted by using the overlaps. The realization of the network, the target, and the input patterns are identical across panels, whereas the noise strength s is increased from the upper to the lower panels. D: The success rate and the normalized residence time at each pattern are plotted against the noise strength s in (i) and (ii), respectively. The success rate is defined in the same manner as in Fig 7A, and calculated across twenty-five realizations of networks. The normalized residence time is defined in the text, and obtained only from the successful recalls in the twenty-five realizations. The dots in (ii) indicate the durations of different realizations.

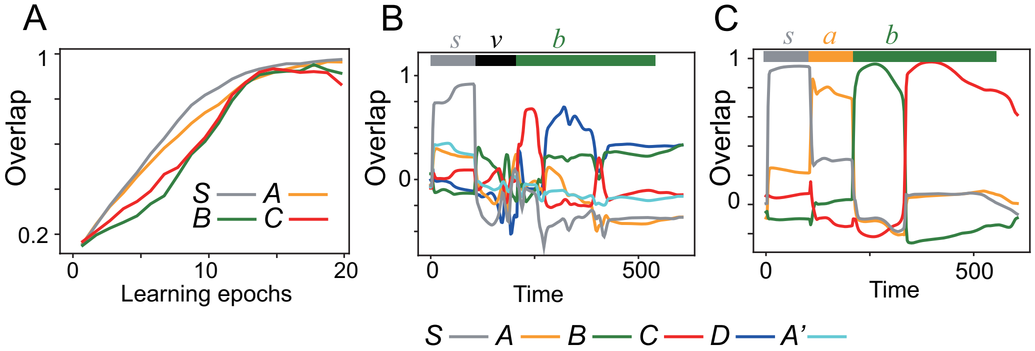

Fig.S 4. A: Learning performance of the first sequence. The performance is measured by the average value of the overlaps of the fast dynamics with patterns in the first sequence (S, A, B, C). These values are obtained from 20 realizations of networks. Which of the patterns is used for the overlap is shown in a different color in the panel. B and C: The fast dynamics in the recall after the learning of the second sequence (S, A', B). The recall processes are shown by using the overlaps with patterns S, A, B, C, D, A' in different colors as indicated at the bottom of the panels. In B, the recall in response to (s, v, b) is plotted, whereas that in response to (s, a, b) is plotted in C. v is a random pattern that is not learned.

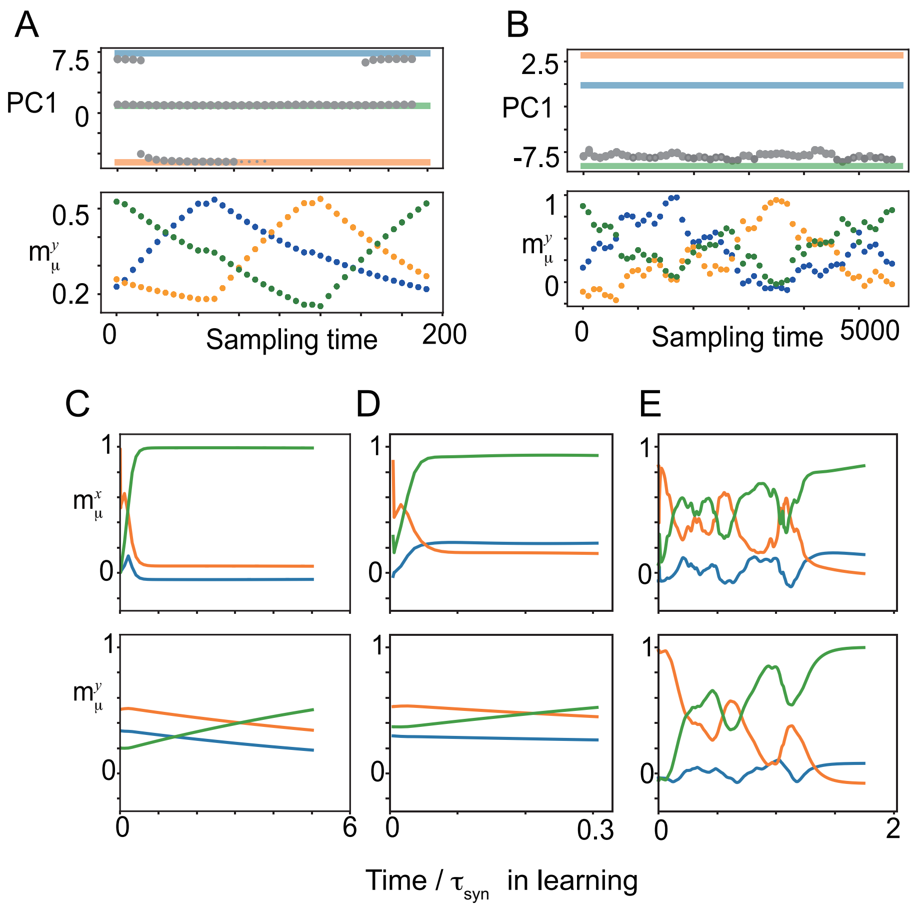

Fig.S 5. A and B: The bifurcation diagrams of the fast variables with quenched $\boldsymbol{y}$ are shown for $(\tau_y, \tau_{syn}) = (100, 10)$ and $(100, 1000)$, respectively. The neural dynamics for $(K, M) = (1, 3)$ are used. These diagrams are plotted in the basically same manner, as shown in Fig. 2B. At a different point from the analysis in Fig. 2B for $\tau_y = 100, \tau_{syn} = 100$, the slow variables are sampled from the trajectory in the final learning step of the sequence (namely, after learning the sequence nineteen times), because all targets do not appear in the recall process. The fixed points are plotted as circles, and colored lines represent the locations of the target 1 (blue), 2 (orange), and 3 (green) by projection onto the 1st principal components in the upper panels by the principal component analysis. In the lower panels, sampled $\boldsymbol{y}$ from the learning process are plotted by using the overlaps with the same color codes as in Fig. 7B. C-E: The neural activities of $\boldsymbol{x}$ and $\boldsymbol{y}$ during learning are plotted by using their overlap with the targets in top and bottom panels, respectively. As examples of the learning process for different timescale-relationship, the neural activities for learning $\boldsymbol{\xi}_3^1$ on the final learning epoch are adopted for $(\tau_y, \tau_{syn}) = (100, 10)$ and $(100, 1000)$ and shown in C and E, while that for $(\tau_y, \tau_{syn}) = (100, 100)$ is plotted in D for reference. Here, the horizontal axis represents the time normalized by τ_{syn}.
